# Universal RNA *Secondary* Structure Insight Into Mosquito-Borne *Flavivirus* (MBFV) *cis-*Acting RNA Biology

**DOI:** 10.3389/fmicb.2020.00473

**Published:** 2020-03-27

**Authors:** Miao Zeng, Yanping Duan, Wei Zhang, Mingshu Wang, Renyong Jia, Dekang Zhu, Mafeng Liu, Xinxin Zhao, Qiao Yang, Ying Wu, Shaqiu Zhang, Yunya Liu, Ling Zhang, Yangling Yu, Shun Chen, Anchun Cheng

**Affiliations:** ^1^Research Center of Avian Disease, College of Veterinary Medicine, Sichuan Agricultural University, Chengdu, China; ^2^Institute of Preventive Veterinary Medicine, College of Veterinary Medicine, Sichuan Agricultural University, Chengdu, China; ^3^Key Laboratory of Animal Disease and Human Health of Sichuan Province, Chengdu, China

**Keywords:** mosquito-borne *flavivirus*, *cis-*acting RNA, RNA secondary structure, RNA-binding proteins, RNA biology

## Abstract

Mosquito-borne flaviviruses (MBFVs) spread between vertebrate (mammals and birds) and invertebrate (mosquitoes) hosts. The *cis-*acting RNAs of MBFV share common evolutionary origins and contain frequent alterations, which control the balance of linear and circular genome conformations and allow effective replication. Importantly, multiple *cis-*acting RNAs interact with *trans-*acting regulatory RNA-binding proteins (RBPs) and affect the MBFV lifecycle process, including viral replicase binding, viral RNA translation-cyclisation-synthesis and nucleocapsid assembly. Considering that extensive structural probing analyses have been performed on MBFV *cis-*acting RNAs, herein the homologous RNA structures are online folded and consensus structures are constructed by sort. The specific traits and underlying biology of MBFV *cis-*acting RNA are illuminated accordingly in a review of RNA structure. These findings deepen our understanding of MBFV *cis-*acting RNA biology and serve as a resource for designing therapeutics in targeting protein-viral RNA interaction or viral RNA secondary structures.

## Introduction

The members of the mosquito-borne *flavivirus* (MBFV) replicate in vertebrates and/or mosquitoes and include a large number of zoonotic pathogens that are divided into eight groups with genetic and serotype divergences: the Japanese encephalitis group (JEVG), Ntaya virus group (NTAVG), Kokobera virus group (KOKVG), Aroa virus group (AROVG), Lammi virus group (LAMVG), YFV group (YFVG), Spondweni virus group (SPOVG) and Dengue virus group (DENVG) (Benzarti et al., 2019). In terms of the naturally infected vertebrate hosts, the majority of MBFV groups (MBFVGs) demonstrate a strong preference for primates (Fernandez-Sanles et al., 2017), while birds act as the primary hosts for JEVG and NTAVG (bird-adaptable MBFVGs), and they serve as amplifying or bridging hosts that have the risk of transmitting the virus to humans (Maharaj et al., 2018).

The MBFV genome is flanked by terminal *cis-*acting RNAs that contain a number thermodynamically stable and shape-conserved structural elements. At the same time, sequence and structural polymorphisms of *cis-*acting RNAs are observed across MBFVGs. The 5′-terminal *cis-*acting RNA contains two stem-loops (SL; 5′SLA and 5′SLB), and the adjacent hairpin in the capsid-coding region (5′cHP) (Gebhard et al., 2011) (Supplementary Figure S1a). The 3′-terminal *cis-*acting RNA can be divided into three independent domains (Gritsun and Gould, 2006a, b, 2007) (Supplementary Figure S1a): Domain I features a variable region (3′VR) located downstream of the terminal codon of the coding region, where the divergence is primarily concentrated, consisting of long deletions, insertions, sequence repeats. Domain II displays two dumbbell-like secondary structures (3′DB1 and 3′DB2). Domain III contains the conserved sequence 3′CS1, a small hairpin (3′sHP) and the 3′SL. Local RNA structures throughout the genome have been computationally predicted by Proutski et al. (1997). Further extensive structural probing analyses have been experimentally performed on *cis-*acting RNAs of several MBFVs (Lodeiro et al., 2009; Wang et al., 2017; Huber et al., 2019). However, the sequence divergence and structure heterogeneity of MBFV *cis-*acting RNA are less-known. With that in mind, prediction of the possible folding of the homologous RNA structure of *cis-*acting RNA of each MBFV was done by the Mfold^1^. Remarkably, *cis-*acting RNAs can be a signal for antiviral or proviral cofactors in a structure dependent manner (Tables 1–3; Chahar et al., 2013; Li et al., 2013, 2014; Phillips et al., 2016; Ward et al., 2016; Chavali et al., 2017; Chiu et al., 2018), which further indicated that RNAs share common evolutionary origins and contain frequent covariations to retain these RNA structures. In this sense, a comparative and thorough analysis on *cis-*acting RNA structures are needed to be conducted as means of identifying possible determinants of MBFV replication and pathogenicity.

**TABLE 1:** Terminal 5′ and 3′ *cis-*acting RNAs and their common co-factor proteins.

*cis-*RNA element	*cis-*RNA binding proteins	Function characteristics	Mechanisms basis	References
5′SLA-TL, 3′SL-TL (JEV)	La protein	Proviral: facilitate replication,	Bind both 3′ and 5′UTR simultaneously stabilizing this structure as well as recruiting NS5 and NS3	Vashist et al., 2009, 2011
5′SLA, 3′SL (WNV)	2′ 5′-oligoadenylate synthetase (OAS1)	Antiviral: repress viral replication	Stimulate the interferon-mediated innate immune response.	Deo et al., 2014, 2015
5′SLB, 3′SL (ZIKV/DENV/WNV)	hnRNP D/AUF-1	Proviral: facilitate replication	Supports cyclization of the viral genome RNA, stimulate NS5 to catalyze RNA synthesis, enables initiation of replication acting as chaperone	Friedrich et al., 2014, 2018
5′UTR, 3′SL(DENV)	NF90/ILF3	Proviral: facilitate replication, RNA transcription and production of infectious virus	Supports cyclization of the viral genome RNA acting as chaperone in a complex of double-strand binding factors (NF90, RHA and NF45)	Gomila et al., 2011
5′UTR,3′CS-SL(DENV4)	La protein	Proviral: facilitate viral replication but not viral translation or entry	Maintain RNA and cyclization structures as nuclear RNA chaperones (JEV); involve in viral replicase (DENV4) and regulate the viral replicase activity. (May serve as a component of translation-to-replication switch mechanism)	Garcia-Montalvo et al., 2004; Vashist et al., 2009, 2011
(-)3′ SL, 5′UTR + /(-) RNA (JEV)	PTB	Antiviral (JEV): repress viral replication	Competitively inhibit NS5 binding to viral negative-strand RNA	Bhullar et al., 2014
5′UTR, 3′UTR (JEV, DENV2)	Far upstream element binding protein 1 (FBP1)	Antiviral: repress translation and viral production	Suppress virus protein expression acting as SG/P body-associated proteins	Chien et al., 2011
5′UTR, 3′UTR (WNV)	Core protein (Cp) (C1-24aa, C80–105aa)	Proviral: RNA cyclization and virus replication	Increase 5′-3′ genomic RNA interactions as a molecular chaperone	Ivanyi-Nagy and Darlix, 2012
5′UTR, 3′UTR (JEV)	DEAD box RNA helicase 3 (DDX3)	Proviral: enhance viral translation and replication	Function as cellular helicase	Li et al., 2014

*For each RNA element, the co-factors, the virus(es) and the step(s) of the affected virus life cycle are indicated. At the same time, the general roles of *cis-*RNA binding protein in regulating viral lifecycle are included.*

**TABLE 2:** Terminal 5′*cis-*acting RNAs and the binding proteins involved in MBFV life cycle.

*cis-*RNA element	*cis-*RNA binding proteins	Function characteristics	Mechanisms basis	Referencess
5′ cap- m7 GpppAmp	Translation elongation factor(eIF4A, E, G, F,B)	Proviral: facilitate translation	Modulate the translation of cellular and viral genome mRNA	Gingras et al., 1999; Harvey et al., 2018
5′ cap- m7 GpppAmp (WNV)	IFIT	Proviral: facilitate translation	Modulate the antiviral effects of IFIT by the cap (m7GpppAmp)	Daffis et al., 2010; Szretter et al., 2012
5′ triphosphates (5′-3P, ZIKV)	RIG-I	Antiviral: recognize nascent transcripts	Lead to interferon secretion	Chazal et al., 2018
5′UTR-ter20nt (WNV)	oligomer (PMO)	Antiviral: inhibit virus replication	Inhibit both the N-7 cap and 2′-OH ribose methylations	Dong et al., 2007
The specific 2nd, 3rd nucleotides in 5′SLA (WNV)	NS5	Proviral: involve in vRNA replication	Involved in N-7 cap methylation	Dong et al., 2007
A minimum 5′UTR-ter20nt and specific 1st and 2nd nucleotides (WNV)		Proviral: involve in RNA replication	Involved in 2′-OH ribose methylation	Dong et al., 2007
The terminal 5′-AGAA-3′ in 5′SLA (DENV)		Proviral: involve in vRNA synthesis	Initiate the *de novo* synthesis of negative-strand RNA	Nomaguchi et al., 2003
5′UTR-ter12nt	NS3	Proviral: facilitate replication	Affect translocation of viral RNA through dynamic and stimulate ATPase activity of NS3	Swarbrick et al., 2017

**TABLE 3:** Terminal 3′*cis-*acting RNAs and the binding proteins involved in MBFV life cycle.

*cis-*RNA element	*cis-*RNA binding proteins	Function characteristics	Mechanisms basis	References
3′UTR (JEV)	DDX5	Proviral: enhance viral translation and replication	Function as cellular helicase	Li et al., 2013
3′UTR (ZIKV)	Musashi-1 (MSI 1)	Proviral: enhance viral replication	Disrupt the binding of MSI 1 to its endogenous targets, involve in cellular pathways, stabilize the viral RNA genome and/or regulate its cyclization or synthesis and	Chavali et al., 2017
3′UTR (DENV2)	Cold shock domain containing protein E1 (CSDE1)	Proviral: translation, synthesis or stability	Modify the vRNA structure and recruit other factors as RNA chaperone activity,	Phillips et al., 2016
3′UTR (DENV)	LSm1	Proviral: enhance replication	Associate with RNA degradation as P-body protein	Dong et al., 2015
3′SL-II/PK1, 3′SL-IV/PK2 (JEV/WNV) 3′SL-I/PK1, 3′SLII/PK2 (ZIKA//DENV)	Exoribonuclease XRN1/2	Proviral: facilitate virus replication	sfRNA inhibit XRN1 activity, stabilize cellular mRNAs, inhibit the antiviral activity of IFN-I and enhance cytopathicity in cells and pathogenicity in mice (inhibit innate immune pathway of insect)	Pijlman et al., 2008; Roby et al., 2014; Gokhale and Horner, 2017; Chen et al., 2018
3′SL-I, 3′SLII (ZIKV)	Fragile X mental retardation protein (FMRP)	Antiviral: repress viral translation	Binds to the ZIKV sfRNA and antagonizes sfRNA	Soto-Acosta et al., 2018
3′VR,sfRNA (DENV2)	Tripartite motif containing 25 (TRIM25)	Repress viral replication	Prevent TRIM25 deubiquitylation and evade the innate immune response	Manokaran et al., 2015
3′VR-xrRNA (DENV4)	quaking protein (QKI)	Antiviral: reduce translation and viral particle production,	Specificially bind to DENV4 but not DENV2	Liao et al., 2018
3′DB (JEV)	Zinc-finger antiviral protein (ZAP)	Antiviral: induces translation repression and RNA degradation	Depend on 3′-5′ RNA exosome-mediated, but not the 5′-3′ XRN1-mediated RNA decay pathway	Chiu et al., 2018
3′UTR (likely DB) (DENV2/YFV17D)	Golgi associated ERI3	Proviral: facilitate viral RNA synthesis	Re-localize to virus replication sites and without affecting RNA stability or translation	Ward et al., 2016
3′DB1, 3′DB2 (DENV2)	DDX6	Proviral: facilitate viral encapsidation and the production of infectious particles	Associate with P-body	Ward et al., 2011; Chahar et al., 2013
A-rich region in 3′DB (DENV)	Poly(A)-binding protein (PABP)	Enhance translation initiation, RNA circularization	Bind to the eIF4G and facilitate the translation initiation complex assembling	Polacek et al., 2009b
3′(-) SL/internal loops/5nt-AU (WNV)	TIAR and TIA-1	Proviral: facilitate virus replication and virus production	Associate with translational repression of cellular mRNA as stress granule protein	Li et al., 2002; Emara et al., 2008
3′CS-SL(DENV)	Polypyrimidine tract-binding protein (PTB)	Proviral: enhance virus translation and replication	Accumulate RNA and involve in the RC, maintain RNA and cyclization structures as nuclear factors, RNA chaperones	Agis-Juarez et al., 2009; Anwar et al., 2009
3′UTR (DENV)	Heterogeneous ribonucleoprotein (hnRNP) C1/C2	Proviral: enhance virus replication not translation	Form the viral ribonucleoprotein (RNP) complexes	Phillips et al., 2016
3′SL (DENV4/DENV2)	eEF-1A	Proviral: facilitate virus replication	Function as an RNA helicase and reduce sphingosine kinase 1 activity	De Nova-Ocampo et al., 2002; Carr et al., 2013
3′SL (DENV)	NF-KB2	Proviral: enhance virus replication	Stabilize RNA and facilitate RNA transcription and transport	Lei et al., 2011
3′SL-S2, S3 (DENV2)	Y Box-binding protein-1 (YB1)	Antiviral: inhibit translation Proviral: facilitate virus production	Play an antiviral role during infection and form the viral ribonucleoprotein (RNP) complexes	Paranjape and Harris, 2007; Phillips et al., 2016
3′SL, (-)3′UTR ter1-160nt (JEV)	Glyceraldehyde-3-phosphate dehydrogenase (GAPDH)	Proviral: facilitate virus replication	Change subcellular localization of GAPDH and promote asymmetric RNA replication, colocalize with NS5 via viral RNA	Yang et al., 2009
3′SL-S2, 3′SL-TL(WNV)	eEF1A	Proviral: supports negative strand RNA synthesis but not translation	Facilitate an interaction between the 3′ end of the genome and the RC as an RNA helicase	Davis et al., 2007
3′SL (DENV1)	NS3	Proviral: facilitate RNA synthesis	Form the replicase	Cui et al., 1998

Beyond local RNA structure in the linear genome, the long-range 5′-to-3′ circularized *cis-*acting RNA (5′-3′cirRNA) has been shown to position the flaviviral RNA-dependent RNA polymerase (RdRp) close to the transcription start site (Filomatori et al., 2011). When no viral proteins are present, the 3′SL in the linear genome may enhance viral translation at the initiation stage, possibly via an interaction with the cellular translation machinery (Table 1; Holden and Harris, 2004). As the amount of viral protein accumulates to form the viral replicase, 5′SLB, 3′sHP, and the lower stem of 3′SL of the linear conformation need to unfold to permit the 5′-3′cirRNA formation via the reversed complementary sequences, such as 5′UAR/3′UAR (upstream AUG region), 5′DAR/3′DAR (downstream AUG region) and 5′CS/3′CS (conserved sequence) elements (Wang et al., 2017) (Supplementary Figures S1a,b). It’s believed that the 5′UAR-flanking stem (5′UFS) functions as a riboswitch by dictating NS5 recruitment and vRNA cyclization. NS5 specifically recognizes and binds to 5′SLA and 5′UFS of the linear genome. Following that, the vRNA circularization would start and highly structured 5′UFS would unwind (Liu et al., 2016). Concurrently, it would favor NS5 to transfer the 3′SL, hence properly positioning the polymerase for initiating the negative-strand RNA synthesis, which is the first step of progeny propagation. In the replicase complex, the multifunctional NS3 protease/helicase is suggested to be involved in folding or unfolding of vRNA structures stimulated by NS5 (Xu et al., 2019). In addition to the NS5 replicase, viral as well as cellular proteins have been suggested to support *flavivirus* RNA replication. The circularization mechanism is prerequisite for vRNA synthesis, nevertheless, several complementary elements show low conservation rates amongst MBFVGs. Particularly, the synergy and respective functions of the complementary elements involved in 5′-3′cirRNA formation remain to be clarified.

Here optimal RNA secondary structures were predicted, and consensus secondary structures of the core *cis-*acting RNA were modeled. The homology and polymorphism of secondary structures of each MBFVG *cis-*acting RNA element were characterized by group. Considering that the current MBFV *cis-*acting RNA biology isn’t a complete mechanism system, we also describe the *cis-*acting RNA biology and pinpoint possible functional determinants of the *cis-*acting RNA accordingly in a review of MBFV RNA structure (Tables 1–3). Antiviral and vaccine strategies that target protein-viral RNA interaction or viral RNA secondary structures are promising.

### MBFV 5′-3′cirRNA

#### Similarity and Difference on MBFV 5′-3′cirRNA Complementary Sequences

**FIGURE 1:**
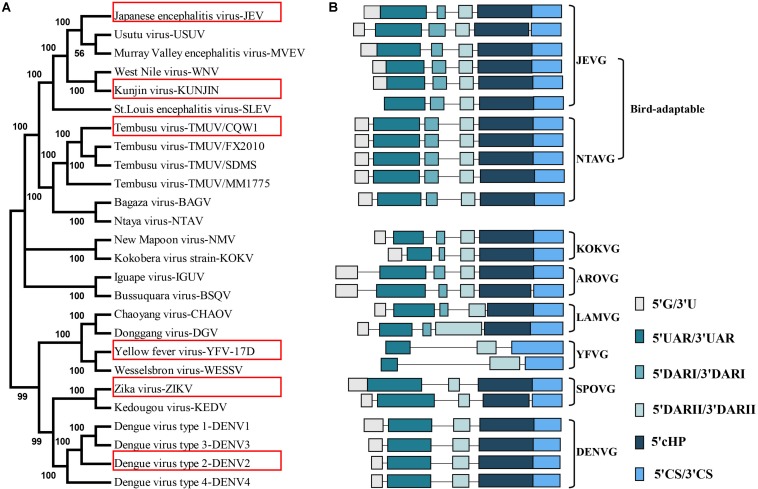
Mosquito-borne flaviviruse genome phylogenetic analysis and schematic depiction of the common structural architecture of 5′-3′cirRNA. **(A)** The maximum-likelihood phylogenetic tree of the MBFVs. Based on the complete-coding-sequence nucleotide alignments, the phylogenetic tree was constructed in MEGA6 with the representative MBFVs in the red box. **(B)** Structure schematic of the cyclization elements, highlighting the eight MBFVGs and the bird-adaptable MBFVG. The cyclization schematic diagram of NTAV species is lacking because its 3′*cis-*acting RNA isn’t registered in NCBI. Each element is proportionally scaled and presents as corresponding colors.

Mosquito-borne flaviviruses have evolved into eight distinct groups with profound differences in their genome sequences (Figure 1A). Although *cis-*acting RNAs typically show large variance both in length and sequence composition, rendering them inconsistently aligned and ill-suited for phylogeny reconstruction. This genome-based phylogenetic classification is in good agreement with 5′-3′cirRNA secondary structure clustering, mainly because all MBFVs share a common circularized architecture (Figure 1B). All the 5′-3′cirRNAs are maintained by an intact 5′SLA, 5′cHP and incomplete 3′SL at the termini (Supplementary Figures S1a,b). Moreover, the representative 5′-3′cirRNAs of bird-adaptable MBFVG possess shorter single-stranded spacer neighboring the 5′SLA (Supplementary Figure S1b). At least three pairs of complementary sequences have been shown to participate in the 5′-3′cirRNA composition, i.e. the 5′CS/3′CS, 5′DAR/3′DAR and 5′UAR/3′UAR elements (Figure 1B). More recently, a new pair of complementary sequences, 5′C1/3′-DB1, has been identified by disassembling the hairpin residing in the 5′cHp and 3′DB1 (De Borba et al., 2015). Notably, except the YFVG, SPOVG and DENVG, all the rest of MBFVGs share two putative DAR regions (DARI, DARII) (Figure 1B and Supplementary Figure S1b). It’s worth mentioning that the 5′G/3′U rich duplex element is preserved as diverse sizes across MBFVGs but lacking for the YFVG (Figure 1B and Supplementary Figure S1b), whereas the 5′G/3′U element in the 5′-3′cirRNA hasn’t been previously proved and much concerned (Basu and Brinton, 2011).

**FIGURE 2:**
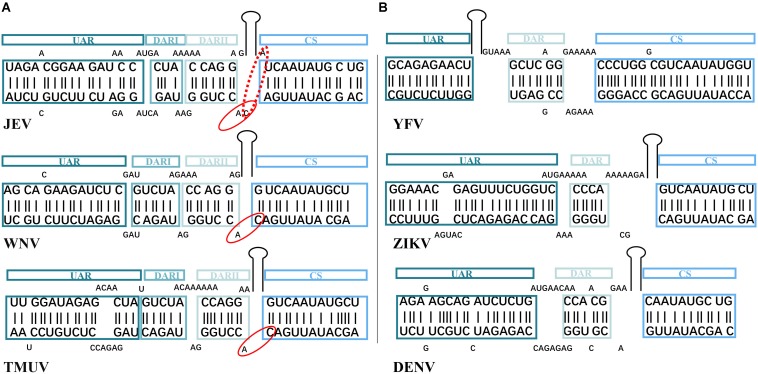
Similarity and difference on complementary sequences of representative MBFVs. The corresponding color for each structural motif (5′UAR/3′UAR, 5′DARI/3′DARI, 5′DARII/3′DARII, and 5′CS/3′CS elements) is maintained as in Figure 1 representation. **(A,B)** Are the bird-adaptable and none-bird adaptable *flavivirus* groups, respectively. The neighboring nucleotides of the conserved 5′CS/3′CS are inconsistent in the bird-adaptable MBFVs. Owing to the unpaired A/C in the dashed ovals of JEV, the dinucleotides in solid ovals distinguish the JEV from other bird-adaptable MBFVs.

Owing to the inconsistent neighboring bases close to the highly conserved 5′CS/3′CS, the first base-pairing of 5′CS/3′CS is undefined in MBFVGs (Figure 2). Both the 5′CS/3′CS and 5′DARII/3′DARII elements in bird-adaptable MBFVGs are highly conserved in sequence and structure (Figure 2A), while the YFVG 5′CS/3′CS is typically longer (Figure 2B, Supplementary Figure S2). Structural polymorphisms of 5′DARI/3′DARI were observed among the bird-adaptable MBFVGs even though the 5′DARI and 3′DARI sequences are relatively conserved. For instance, the initial base-pairing of 5′DARI/3′DARI is also inconsistent resulting from the uncertain neighboring matchings (Figure 2A and Supplementary Figure S2). Interestingly, both the topology and sequence of 5′DAR/3′DAR in none-bird-adaptable MBFVGs closely resembles 5′-3′DARII in the dual-DAR MBFVGs (Figure 2B). Last but not least, the 5′UAR/3′UAR are strikingly divergent with different quantities and sizes of bulges even in the same MBFVG (Figure 2). Additionally, the internal unmatched base-pairings in 5′UAR/3′UAR are less conserved in 5′-3′cirRNA and tend to participate in the local secondary structure in the linear viral genome.

#### Efficient and Alternative 5′-3′cirRNA Dominates *MBFV* vRNA Synthesis

During the MBFV cyclization process, the dynamic 5′-3′cirRNA initiates from the 5′CS/3′CS duplex formation, then extends the initial interaction by the 5′DAR/3′DAR, and assists the interaction of the 5′UAR/3′UAR element which would result from unwinding the bottom of terminal 3′SL (Polacek et al., 2009a; Friebe et al., 2011). Moreover, a sequence present downstream of the 5′CS element in the capsid-coding sequence called “downstream CS”(dCS) impacts genome circularization by modulating the topology of 5′*cis-*acting RNA (Friebe et al., 2012). Earlier reports showed that 5′CS/3′CS element was demonstrated as the most stable interactions in both the DENV and ZIKV viruses (Huber et al., 2019). Under stringent cellular conditions, the isoform p45 of host protein AUF1 (also termed hnRNP D) destabilizes 5′SLB and the 3′SL, thereby exposing the 5′UAR element and promoting vRNA cyclization, and positively regulates the replication of WNV, DENV and ZIKV (Table 1; Friedrich et al., 2014,2018). It is conceivable that depletion of AUF45 inhibits virus replication and infection. In another report, the core protein was found to induce a dramatic acceleration in the 5′-3′cirRNA annealing *in vitro* (Ivanyi-Nagy and Darlix, 2012). Upon core protein chaperoning, it might stimulate intramolecular RNA rearrangements or intermolecular 5′-3′cirRNA interactions without ATP consumption. Indeed, binding of the cellular and viral protein to 5′-3′cirRNA of MBFVs seems to be a viable mechanism for regulating the 5′-3′cirRNA affinity which is critical for the initiation of negative-stranded RNA through NS5 recruitment to 3′*cis-*acting RNA (Table 1; Garcia-Montalvo et al., 2004; Vashist et al., 2009, 2011; Gomila et al., 2011; Bhullar et al., 2014).

Deletion of the complete 3′CS could partially restore 5′-3′cirRNA conformation and rescue the lethal WNV through compensatory mutations on the 5′UAR/3′UAR and 5′DAR/3′DAR elements (Zhang et al., 2010). Furthermore, the effect of the individual base-pairing on replicons varied with their position (Suzuki et al., 2008), mutations on the central position of 5′CS/3′CS sequence have negligible effect on replication, whereas base-pairings in the terminal position severely affect viral replication. In this case, a number of adjacent 5′CS/3′CS mismatching combinations were also rescued by a second site mutation that created additional base-pairings on the internal genomic side of the 5′CS/3′CS element (Basu and Brinton, 2011). Interestingly, mutations in the DENV single 5′DAR disrupts the 5′-3′cirRNA interaction, whereas mutant 3′DAR still retains alternative 5′-3′cirRNA conformation (Friebe and Harris, 2010). Likewise, mutations in the central three nucleotides of the WNV 5′-3′DARII decreased but not disrupted the affinity of 5′-3′cirRNA (Friebe et al., 2011). As such, the abrogated viral replication may directly result from the decreased affinity of the 5′-3′cirRNA, otherwise mediated by the corresponding 5′DARII nucleotides and/or 3′DARII structures in the linear viral genome. Consistently, the 5′DARII is single-stranded, while the 3′DARII is involved in the 3′sHP formation in the linear viral genome. Both the complementarity 5′DAR/3′DAR element and the formation of 3′sHP and 3′SL are required for vRNA replication of MBFVs. In most of the 5′UAR/3′UAR cases, a single mutation disrupting complementarity can greatly compromise vRNA synthesis, and compensatory mutations could potentially reestablish alternative 5′UAR/3′UAR element, then modulate vRNA synthesis but not viral translation (Alvarez et al., 2005b, 2008). Given that the internal unmatched base-pairing in 5′-3′cirRNA is less conserved and tends to participate in the local secondary structure of the viral linear genome, it is likely that these nucleotides would regulate stability of the 5′-3′cirRNA.

### Promoter and Enhancer of 5′*cis-*Acting RNA

#### RNA-Binding Proteins (RBPs) Direct 5′SLA Promoter-Dependent vRNA Synthesis

**FIGURE 3:**
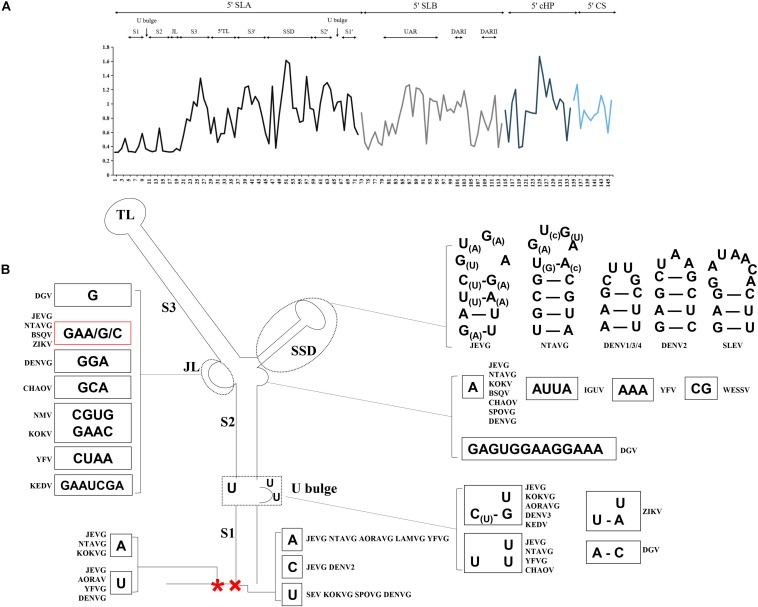
**(A)** Mean relative evolutionary rates are shown for each site of the 5′*cis-*acting RNA. The analysis involved 25 nucleotide sequences from all the MBFVGs, spanning the 5′SLA, 5′SLB, 5′cHP and 5′CS. Evolutionary analyses were conducted in MEGA6. These rates are scaled such that the average evolutionary rate across all sites is 1. This means that sites showing a rate <1 are evolving slower than average, and those with a rate >1 are evolving faster than average. **(B)** MBFV structured promoter 5′SLA features divergent nucleotides. The 3rd A/U nucleotide (*) undergoes a nucleotide substitution, and the 4th nucleotide (×) shows sequence and structure divergences even in the same JEVG or DENVG.

Mosquito-borne flaviviruse genomic RNA begins as a Y-shaped-like long stem structure (termed the 5′SLA) that comprises three stems (S1, S1, and S3) characterized by the U bulge, top loop (5′TL) and side structure domain (SSD). Although the size and shape of the 5′SLA are highly conserved, sequence conservation isn’t restricted to the local stems and loop regions (Figure 3 and Supplementary Figure S3). For instance, three stem regions are preserved though a number of covariations, not least because S3 can tolerate large variations on the length and sequence (Lodeiro et al., 2009). That is, NTAVG and SLEV have insertions resulting in overall lengthening of the S3 region (Supplementary Figure S3). Such substantial sequence divergences likely contribute to 5′SLA specific binding ability for RBPs. At the forefront of the 5′SLA promoter are the well-conserved dinucleotide “AG” in all MBFVs except the KEDV and DGV. However, nucleotides at the third site extensively undergo an 3rd A/U nucleotide substitution, and the 4th nucleotides seem to show sequence and structure divergences even in the same JEVG or DENVG (Figure 3B and Supplementary Figure S3). The junction loop between S2 and S3 exhibits irregular in sequence and structure among MBFVGs. Nevertheless, the bird-adaptable group is characterized by a relatively regular junction loop “GAA/G” (Figure 3 and Supplementary Figure S3). The structurally conserved 5′TL is less variable in length than the loop region of SSD (SSL). The SSD that prominently sticks out from the stem backbone demonstrates extensive heterogeneity in size, sequence and secondary structure (Figure 3 and Supplementary Figure S3). Furthermore, a single-isolated “A” nucleotide is located aside SSD, which exclusively belongs to the bird-adaptable groups as well as DENVG and CHAOV.

The 5′m7GpppAmpN2 cap (5′cap) is absolutely conserved in MBFVGs, which enables translation through the canonical cap-dependent translation initiation (Zhao et al., 2015). The addition of 5′cap of vRNA is mediated by the methyltransferase activity of NS5 in combination with nucleotide triphosphatase activity of NS3 (Issur et al., 2009; Zhao et al., 2015). Under these conditions the forefront nucleotides are specifically recognized and regulated by the cap-binding eIF4E protein (Gingras et al., 1999; Lloyd, 2015; Harvey et al., 2018; Table 2). As *flavivirus* NS5 RdRp contains an ATP-specific priming site, which imposes a strong preference for the *de novo* synthesis using a dinucleotide “AG” primer (Nomaguchi et al., 2003; Zhao et al., 2015). The initially terminal 5′-AGAA-3′ is the most optimal template for DENVG RdRp (Nomaguchi et al., 2003). DENV NS3 were previously shown to affect the translocation of vRNA through dynamic interactions with nucleotides at positions 4th U and 5th G of the 5′SLA (terminal sequence 5′-AGUUGUUAGUCU-3′). Residuals D290 and R538 of DENV NS3 also have specific interactions with the 2nd G and 5th G, respectively, Swarbrick et al. (2017). The presence of 2nd G and 5th G presumably drives a molecular switch of NS3 and leads to significantly higher activation of ATPase activity of NS3. Functional analysis of S1 and S2 of 5′SLA demonstrated that disruption of the stems abolished vRNA replication. Genome-length viral RNAs with reconstitution of these stems replicated at a moderately lower rate and generated revertants or second-site mutations upon passaging (Filomatori et al., 2006; Li et al., 2010; Liu et al., 2017). WNV NS5 binds specifically to S1 region (Dong et al., 2008), and requires distinct RNA elements within the S1 for two successive cap methylations (Dong et al., 2007). Beyond that, both the 5′TL and SSL are involved in specific NS5 binding (Table 1). However, such binding determinants were considered to be necessary to promote NS5 polymerase activity but not the prior NS5 binding process (Filomatori et al., 2011). Despite the diversity on SSD, its stable secondary structure was found to be essential for infectivity (Dong et al., 2008; Li et al., 2010; Zhao et al., 2015). Notably, the structurally stable SSD is also sensitive to oligomer (PMO), which is a key antiviral molecular (Dong et al., 2007). Neither the deletion of U bulge nor GAA/G loop alter RdRp binding and activity (Filomatori et al., 2011), whereas it’s proposed that they must interact with other proteins required for viral replication (Table 1).

Interestingly, the 5′cap of viral RNA functions to subvert innate host antiviral responses through escape of IFIT-mediated viral suppression (Daffis et al., 2010; Szretter et al., 2012). In addition, it has been hypothesized that the double-stranded stem of this 5′SLA, which is located several nucleotides away from the 5′ triphosphates (5′-3P), may act a potent agonist and lead to interferon secretion in infected cells (Deo et al., 2014, 2015; Chazal et al., 2018). During replication, RIG-I must bind the 5′-3P of nascent vRNA before capping, which consists of releasing the terminal phosphate from the 5′-3P of the (+) strand by the viral NS3 protein (Cui et al., 1998; Klema et al., 2015). Such recognition by RIG-I of one of the most conserved elements within the vRNA would facilitate virus immune escape.

### Group Structure-Specific 5′SLB Dictates NS5 Recruitment and vRNA Cyclization

**FIGURE 4:**
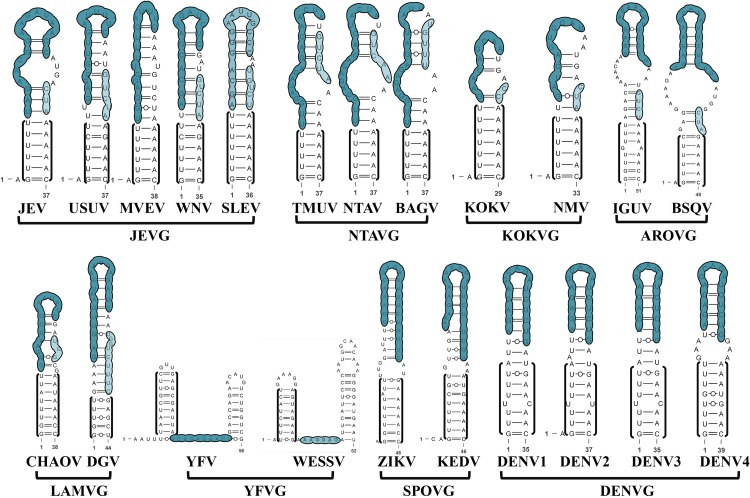
The secondary structure of 5′SLB from eight MBFVGs is group-specific. The 5′UFS base-pairing is indicated at the bottom of 5′SLB in the black bracket. The nucleotides that participate in the MBFV 5′UAR and 5′DAR are labeled in respective colors as above.

The top region of 5′SLB contains the translation initiation codon and projects structure-specificity in each MBFVG (Figure 4). Therefore, we reasonably speculate that some selection pressure can contribute to the top part of 5′SLB. At the bottom of 5′SLB, the oligo(U) region of MBFVG forms a canonical duplex with complementary sequences in or near the 5′UAR sequence and vRNA translation start region, which is designated as the 5′UFS. Because the local folding pattern of 5′SLB and 5′-3′cirRNA in the YFVG are strikingly different from other MBFVGs, the corresponding 5′UFS in YFVG is recognized as the AU-rich hairpin (Figure 4 and Supplementary Figure S1b). The structural 5′UFS unwinds in response to 5′-3′cirRNA, leading to the decreased NS5 affinity for the 5′*cis-*acting RNA and NS5 transferring to the 3′*cis-*acting RNA. However, the G-C base-pairings might increase the potential stability of 5′UFS, as in WNV and USUV (Figure 4). Further research also showed that stabilizing 5′UFS impaired both vRNA cyclization and replication (Liu et al., 2016). An unstructured 5′DARII decollates the 5′SLB and 5′cHP, specially preserved in the bird-adaptable group, KOKVG and AROAVG (Supplementary Figure S2).

### 5′cHP Facilitates vRNA Translation Initiation Selection as Well as vRNA Synthesis

**FIGURE 5:**
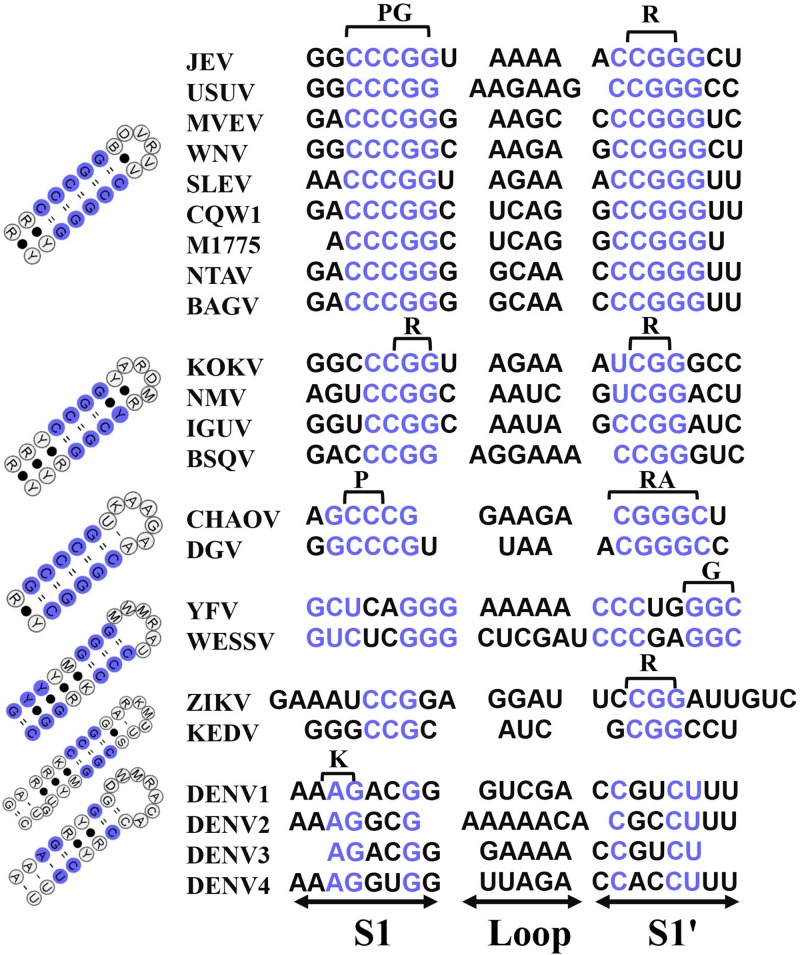
Comparison of the homologous 5′cHP elements among MBFVGs. The nucleotide alignments of 5′cHP regions are in the left. The conserved nucleotides on the stem of each group are highlighted in blue. The abbreviations of amino acids determined by the conserved nucleotides are annotated. The 5′cHP models of the closely phylogenetically related MBFVGs are in the left. The commonly used merger base codes were used for construction of the consensus mode (M = A,C; R = A,G; W = A,T; S = G,C; Y = C,T; K = G,T; V = A,G,C; H = A,C,T; D = A,G,T; B = G,C,T; N = A,G,C,T).

For each MBFVG, comparison on stem region of the homologous 5′cHP element reveals varying degrees of nucleotide conservation, regardless of the extremely non-conservative loop sequence (Figures 3A, 5). That is, the position of the conserved nucleotide is MBFVG-specific, and besides that the corresponding conserved nucleotides of the two stems (S1, S2) can precisely be base-paired. Particular for the YFVG, SPOVG and DENVG, these nucleotides varied with MBFVGs. Importantly, the conserved nucleotides determine the key amino acid as well as the stem structure, heralding an evolution restriction of both the RNA structures and key residuals. The 5′cHP’s structure serves primarily to stall the ribosome over the translation initiation codon, with variable sequence on the stem and loop, but its stability and location with respect to the translation initiation codon apparently correlate with translation efficiency (Clyde et al., 2008). On the other hand, the 5′cHP likely stabilizes the overall 5′-3′cirRNA formation or participates in the recruiting cofactors associated with the replicase machinery during vRNA synthesis.

### Duplicated 3′DBs Confer Contrasting Functions and Host Specificity

**FIGURE 6:**
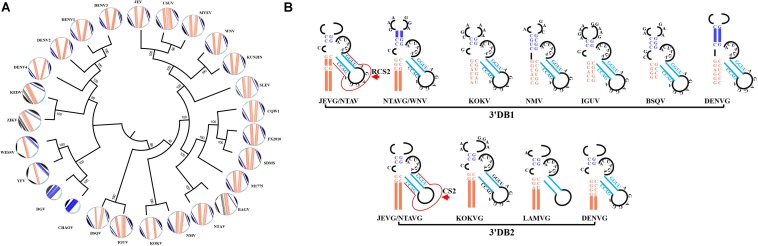
The consensus structure of divergent DB regions within MBFVGs. **(A)** Molecular phylogenetic analysis of the DB sequences. A fan dendrogram indicating the distance between these structures is shown as corresponding circle plot with the sequence and arcs denoting base pairings. The color code for each structural motif is maintained in all the representations. The unrooted phylogenetic tree was built with the maximum likelihood method using MEGA7, based on the alignment of the DB sequences. **(B)** Overview of the bottom part of the consensus dual-DB common to different MBFVs. The highly conserved nucleotides in the individual local secondary structure are annotated in the same color as in panel **(A)**. Meanwhile, the thick colored lines represent the conserved RNA structure in each classified group. The RCS2 and CS2 are marked by arrow in JEVG DB1 and DB2, respectively.

To achieve resilience in host adaptation, 3′DB duplication strategies have been proposed as an evolutionary trait for MBFV. It’s worth mentioning that the single 3′DB is still present in AROAVG, YFVG and SPOVG (Figure 6A and Supplementary Figure S4). The single-stranded linker separates DB1 and DB2 and varies in length and sequence (Supplementary Figures S5, S6). Considering that the top hairpin of 3′DBs should be extraordinary for its similarity in both structure and sequence, only the consensus structures of the bottom part of 3′DBs are constructed and sorted. Additionally, the group-conserved nucleotides are mapped on the homologous structures (Figure 6B). Indeed, nucleotides in the bottom stem of DB1 do not preserve consistency among MBFVGs. The ensuing 5′-CCC-3′ trinucleotide of DB1 allows the first “C” nucleotide to be freely unpaired and consequently constitutes an interior loop, which distinguishes the bird-adaptable groups from the majority of MBFVGs (Figure 6B and Supplementary Figure S4). Unlike DB1, the structure and the nucleotides in the interior loop of DB2 are instead invariant in most of the dual-DB MBFVGs (Figure 6B and Supplementary Figure S6). Such DB2 elements are more easily clustering than DB1, hinting to a lower evolution pressure. Duplicated 3′DBs are incorporating two repeated conserved sequences (CS2 and RCS2). The pseudoknot formation of TL1/PK2 and TL2/PK1 is promoted by the presence of respective loops TL1/TL2 and complementary pentanucleotide PK1/PK2 as well as RCS2 and CS2 (Supplementary Figure S1a).

There are two main explanations to the presence of dual-DB structures: (a) their putative interaction with dimeric proteins and (b) their requirement to sustain replication in a dual-host system of vertebrate hosts and invertebrate vectors. However, the latter hypothesis is proven. Deletion of DENV DB1 reduces viral replication both in human and mosquito cells. It’s intriguingly that deletion of DENV DB2 was greatly advantageous for mosquito infection, with low impact in human cells. Analysis of viruses obtained from infected mosquitoes showed selective mutations mapped in the DB2 (De Borba et al., 2019). Importantly, DB1 and DB2 differentially modulate viral genome cyclization, the pseudoknot formed within DB2 competes with long-range 5′-3′cirRNA interactions (De Borba et al., 2019). Additionally, the respective contributions of TL1 and TL2 to translation appear unequal: TL1 mutation alone does not have any effect; TL2 mutation has only a modest effect in translation; but translation is reduced in the TL1/TL2 double mutant, indicating that TL1 exhibits a cooperative synergy with TL2 in translation (Manzano et al., 2011). A 30nt-deletion corresponding to TL2 of the DB2 structure attenuates all four DENV serotypes and is currently being tested as a vaccine candidate (Alvarez et al., 2005a; Kirkpatrick et al., 2016). Moreover, a 10nt deletion of the 3′DB of ZIKV has shown a target for viral attenuation (Shan et al., 2017). Although mutations abrogating TL/PK complementarity can imped viral translation and replication. Remarkably, restoration of pseudoknots can rescue the translation level but not replication defects. In contrast to TL1 and TL2, PK1 and PK2 are not absolutely necessary for translation, suggesting its alternative TL receptors within the vRNA. Despite the lack of a poly-A tail, PABP appears to specifically bind to the A-rich sequences flanking the 3′DB structures, where the corresponding binding nucleotides aren’t exactly identical. Such interaction mimics the role of mRNA poly-A tail and presumably stimulates translation initiation (Supplementary Figure S4 and Table 3).

### Viral Replication Shows High Vulnerability to the Overlapping RNA Signals on 3′sHP

**FIGURE 7:**
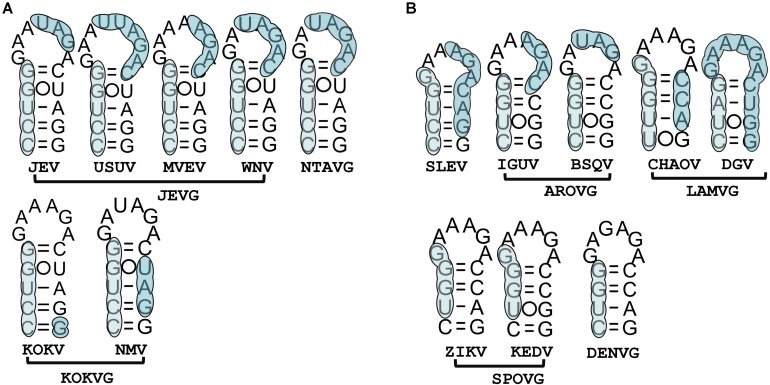
Group-specific 3′sHP characteristic of the overlapping RNA signals. **(A)** MBFVs comprising the bottom two “C-G”. **(B)** MBFVs lacking for the bottom two “C-G”. 3′DARI and 3′DARII elements of the overlapping cyclization sequences for each MBFV are annotated in corresponding color as above.

The 3′UAR and 3′DAR of 5′-3′cirRNA sequences overlap the 3′sHP. In most cases, the highly conserved sequences on the left stem are involved in 5′DARII/3′DARII formation, and the majority of the loop region overlaps with the 3′DARI. Accordingly, the right stems participate in the 3′UAR or 3′DARI in some MBFVs. However, several nucleotides of the loop and right stem only participated in the 3′sHP structure but not the cyclization formation (Figure 7). Hence, we have been wondering if enhancing these bases complementary to the 5′*cis-*acting RNA would affect the viral replication via a more stable 5′-3′cirRNA. The overlapping sequences within the 3′sHP regulate the equilibrium between the two alternative conformations of the genome (Wang et al., 2017).

Except the SLEV of JEVG, the bottom two “C-G” base-pairings of 3′sHP separate JEVG, NTAVG and KOKVG from the other MBFVGs (Figure 7A). In other words, the lacking of “C-G” base-pairings results in a four-base-pair shortage in the non-bird-adaptable MBFVGs (Figure 7B). Studies of the first base-pairing “C-G” of DENVG 3′sHP confirmed that the stability can alter interconversion between the linear and circular conformations of the vRNA during replication (Wang et al., 2017). Alternatively, it has been shown that disruption of the 3′sHP stem abolishes viral replication unless reversion occurs in order to rescue replication via the balance between the alternative conformations (Villordo et al., 2010; Davis et al., 2013). Interestingly, point mutations in the 3′sHP abrogated infection in mosquito cells without affecting replication in mammalian cells (Villordo and Gamarnik, 2013). Remarkably, the loop region of 3′sHP that resembles the typical GNNRA motif, is longer in most members of JEVG, and the sequence is not exactly identical even in the same MBFVG (Figure 7). It has been demonstrated that nucleotides within that loop could form a pseudoknot with corresponding nucleotides in the 3′SL, which has implications for virulence, attenuation and vaccine development (Shi et al., 1996).

### 3′SL Is Endowed With a Virus-Specific Required Sequence (VRS)

**FIGURE 8:**
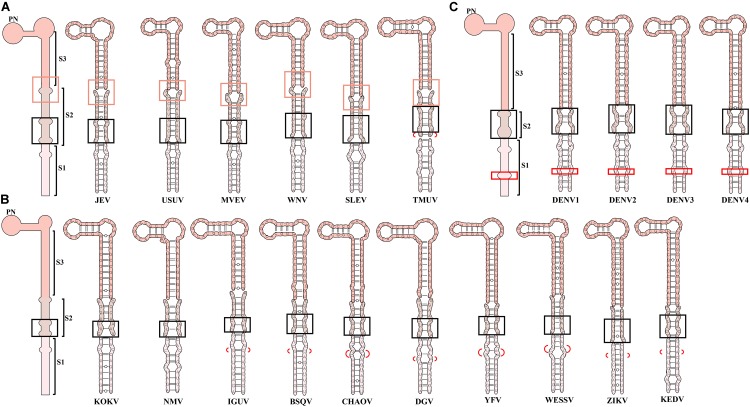
A comprehensive analysis on the 3′SL conformations of MBFVs. Homologous stems and elements are defined in the 3′SL according to the loci of corresponding bulges in **(A)** JEVG, NTAVG. **(B)** KOKVG, AROVG, LAMVG, YFVG, SPOVG, and **(C)** DENVG. A general common structural architecture of 3′SL in the close group is depicted for **(A)** JEVG, NTAVG. **(B)** KOKVG, AROVG, LAMVG, YFVG, SPOVG, and **(C)** DENVG. Three homologous stems (S1, S2, S3) were defined in corresponding colors. The highly conserved homologous bulge and adjacent structure are indicated by the orange box in JEVG and NTAVG. The homologous bulge in the S2 region is indicated by a black box in each group. The extra base-pair insertion in the S1 region is shown in the red bracket or box. Special TMUV/CQW1 insertion marked by the red bracket is in S2 region.

Three homologous stems are defined in the most well-studied 3′SL according to the loci of corresponding bulges (Figure 8). Based on that, the minimal VRS of 3′SL in individual MBFV is easily confirmed using a chimeric genome. The sequence and structure of stem 1 (S1) of the 3′SL are highly conserved across the JEVG, NTAVG and KOKVG (Figure 8A). Notably, the homologous S1 elements are lengthened slightly by extra nucleotides in remaining MBFVGs. The extra base-pair insertion of S1 region is shown in the red bracket or box (Figures 8B,C). Region S1 of MBFVs consists of two conserved base-pairings (U-A, G-C) that are flanked by below and above internal loops. Additionally, the relatively position-fixed C/C bulge in S1 is strictly conserved among JEVG, NTAVG and KOKVG (Figures 8A,B). Region S2 has undergone sequence and structure changes and harbors extra bulges (Figures 8A–C). Specifically, in JEVG, the base-pairings flanked by the terminal bulges of S2 region characterize strictly homologous sequences. Nevertheless, the nucleotides in the middle region of S2 vary in size and sequence (Figure 8A). Totally, the S2 element of DENVG is relatively short and lacking for a bulge region (Figure 8C). In addition, the NTAVG is characteristic of the U-A at the lowest edge of the S2 region (Figure 8A). Despite divergent nucleotide variations, S3 region contains a number of covariations to maintain the stem structure. Except for the YFVG, a stable hairpin comprising three invariable base-pairing and partially conserved nucleotides are observed at the top region of 3′SL (3′TL).

Despite the topological similarity of 3′SL across MBFVs, the analogous 3′SL sequence could not substitute for the original 3′SL to support vRNA replication (Yu and Markoff, 2005). In the DENV2 chimeric genome, containing the WNV 3′SL nucleotide sequences, the VRS is an 11bp segment comprising the majority nucleotides in the S2 region (Zeng et al., 1998). In that case, the two specific bulges of S2 region are critical for viable DENV. Further analysis on the replication phenotypes of WNV chimeric genome has revealed that a bulge within the top portion (S2 and S3) in the 3′SL is essential for WNV replication. In addition, the introduction of a second bulge into the lower part of the long stem of the WNV 3′SL can be an enhancer of replication in cultured mosquito cells but not monkey kidney cells (Yu and Markoff, 2005). Generally, the loci of these bulges are not well conserved among *flavivirus* species. Nonetheless, the integrity of specific bulges is required for vRNA replication. Most likely, bulges are critical sites for binding of viral and cellular proteins to form the *flavivirus* replication complex (Yu and Markoff, 2005; Paranjape and Harris, 2007). Besides that, bulge regions are related to the low temperature transition (Davis et al., 2013). Mutation of the bulges or adjacent nucleotides is detrimental to virus replication (Iglesias and Gamarnik, 2011). In contrast, engineered substitutions within S3 for WNV in the DENV backbone don’t result in loss of infectivity, which is associated with the significant variability of region S3 in MBFV (Zeng et al., 1998).

It’s clear that the NS5 initiates *de novo* vRNA synthesis from the terminal 3rd nucleotide of JEV 3′SL template (Kim et al., 2007). Under such circumstances, it’s notsurprising that SPOVG is not 5′-CU_(OH)_-3′ terminated (Figure 8B). Consistently, the 3′-terminal six nucleotides (5′-GGAUCU(OH)-3′) of the WNV 3′SL exerts discrete effects on RNA replication (Tilgner and Shi, 2004). Mutational analysis of individual nucleotides in the WNV 3′TL showed that the majority were *cis-*acting and that three of the nts (underlined in bold, 5′-CA**C**A**G**UG**C**-3′) were essential for virus replication but did not affect translation (Elghonemy et al., 2005). The 3′SL has been shown to bind both virus-coded and cellular proteins. NS5 apparently recognizes a *cis-*acting element in the 3′(-)SL more efficiently than the one in the 3′(+)SL for the synthesis of plus-strand JEV RNA (Kim et al., 2007). Furthermore, TIA-1 and TIAR interact with 3(-)’SL of WNV and the knockout of these proteins decreases viral titers, implicating that interactions in efficient viral replication (Li et al., 2002; Emara et al., 2008). Actually, ubiquitous host proteins, such as **e**EF-1A, NF-KB2, PTB, GAPDH, PCBP, LSm1 and hnRNP A1, are utilized by several MBFV 3′SL in different ways (Table 1; De Nova-Ocampo et al., 2002; Davis et al., 2007; Agis-Juarez et al., 2009; Anwar et al., 2009; Yang et al., 2009; Lei et al., 2011; Carr et al., 2013; Dong et al., 2015).

### Functional Interplay Between MBFV and Additional 3′*cis-*Acting RNAs

Even though the deletion of the 3′VR decreases DENV2 RNA synthesis in BHK cells, and these recombinant viruses are viable and delayed for replication, it has no effect on RNA replication in C6/36 cells (Alvarez et al., 2005a). Conversely, another report showed that deletion of the 3′VR increased replication in mosquito cells (Villordo et al., 2015). Anyway, RNA structure specialization and duplication in 3′VR are utilized for maintaining host-fitness. The 3′VR of MBFV contains an AU-rich region that is thought to have evolved due to the RdRp stuttering on the UAA stop codon (Gritsun and Gould, 2007) (Supplementary Figures S1a,S6). The length of the AU-rich region varies among MBFVGs, with the WNV and MVEV having the longest AU-rich regions (Gritsun and Gould, 2006b). Importantly, the 3′VR contains a succession of hypervariable exonuclease-resistant structures (xRNAs) structures and plays critical roles in virus-host interactions (Table 3; Pijlman et al., 2008; Roby et al., 2014; Gokhale and Horner, 2017; Chiu et al., 2018; Liao et al., 2018; Soto-Acosta et al., 2018).

Upon *flavivirus* infection, accumulation of viral subgenomic flaviviral RNAs (sfRNAs) is observed, which are associated with viral replication, pathogenesis and cytopathicity (Chapman et al., 2014). Mechanistically, sfRNAs are induced by incomplete degradation of viral gRNA by stalling 5′-3′ exoribonuclease Xrn1 at xrRNAs, including different types of stem-loop (SL) and DB elements (Chapman et al., 2014; Chen et al., 2018; Macfadden et al., 2018). These structures efficiently stall Xrn1 from progressing through from the 5′-terminal direction, thus protecting the downstream RNA from degradation. In mammalian cells, sfRNAs have been shown to globally inhibit inflammatory gene expression and dampen the cellular type I IFN response (Moon et al., 2012; Schuessler et al., 2012). On the other hand, sfRNAs are described to impair the innate immune response by targeting of cytoplasmic pattern recognition receptors (PRRs). High levels of sfRNAs of particular DENV strain would directly bind the TRIM25 in a sequence specific fashion (Manokaran et al., 2015), thus impairs the ubiquitination of RIG-I and thereby its activation. Furthermore, it’s previously proposed that sfRNAs may inhibit interferon-stimulated genes (ISGs) translation through binding of G3BP1, G3BP2, and CAPRIN1 (Bidet et al., 2014). Interestingly, sfRNAs are believed to represent the smallest fragments of DENV RNA that can be replicated during an infection and might influence DENV transmission (Li and Aaskov, 2014).

## Conclusion

Mosquito-borne flaviviruse *cis-*acting RNA exemplifies a paradoxical twist in the homology and polymorphism of RNA sequence and structure, highlighting the potential implications of *flavivirus* evolution and diversification in successful infection. The four large *flavivirus* clusters, MBFVs, TBFVs, no-known-vector flaviviruses (NKVs) and insect-specific flaviviruses (ISFVs), possess differences in their host ranges. On one hand, structure organization and regulation mechanism of *cis-*acting RNA are more restricted to MBFVGs. On the other hand, the dynamics of host-virus interaction drive the heritable genetic diversity and structural polymorphisms on *cis-*acting RNA of MBFVGs.

We review the feature and biology of *cis-*acting RNAs of MBFVGs as follows: (I) MBFVG divergent 5′-3′cirRNAs regulate MBFV replication in an alternative conformation (II) MBFVG 5′*cis-*acting RNA compromises structurally homologous promoter and enhancer despite the low sequence identity (III) 3′*cis-*acting RNA has shown apparently group-specific elements exemplified by 3′DB and 3′sHP structure (IV) Structurally heterogenous 3′SL is endowed with a critical VRS. Additionally, sfRNA can regulate the host cell upon infection. Combining structural and sequence analysis, a large amount of structural heterogeneity between the bird-adaptable and non-bird-adaptable groups is observed. After that, further experiments are necessary to clarify the molecular biology of these structural differences across MBFVGs.

## Author Contributions

MZ conceived and wrote the manuscript, and made the figures. YD and WZ read and revised the manuscript. MW, RJ, DZ, ML, XZ, QY, YW, SZ, YL, LZ, YY, and AC contributed materials and analysis tools. SC contributed to figure modification and revised the manuscript. All authors read and approved the final manuscript.

## Conflict of Interest

The authors declare that the research was conducted in the absence of any commercial or financial relationships that could be construed as a potential conflict of interest.

## Footnotes

1 http://unafold.rna.albany.edu/?q=mfold

## Supplementary Material

FIGURE S1 **(a)** Linear models of MBFV *cis-*acting RNA and the RNA-acting binding proteins (RBP) for MBFV. Using JEVG as model, the proposed secondary structures of the core *cis-*acting RNA represent the typical MBFV genomic RNA. Meanwhile, the conformations of individual *cis-*acting RNA secondary structure are annotated. **(b)** Summary of the representative MBFV 5′-3′cirRNA models. The minimum-free-energy structure is adopted. Colored lines indicate the interacting RNA elements involved in genome cyclization. The same color is used for equivalent structures in different MBFVGs.

FIGURE S2 Comparison of the nucleotide sequences of 5′-3′cirRNA in MBFVs. The different colors denote the indicated inverted complementary sequences. Above the multiple alignments, the secondary structure of JEVG is shown using an arc plot.

FIGURE S3 Sequence and secondary structure of MBFV 5′SLA. **(a)** Comparison of the nucleotide sequences of the MBFV 5′SLA. The multiple sequence-alignments are annotated according to the homologous structures. The S1/2 and S1/2′ are complementary base pairings, and the (^∗^) and (×) markers represent the divergent nucleotides. **(b)** Annotated homologous RNA secondary structure on 5′SLA across MBFVGs. The red dots and arrow in NTAVG indicate the nucleotide variations and insertion compared to the CQW1 strain, respectively. The RNA secondary structure was predicted online by Mfold (http://unafold.rna.albany.edu/?q = mfold).

FIGURE S4 The conserved patterns of structural homology of dual-DB. Divergent nucleotides of group-specific DBs in dual-DB MBFVGs are mapped. The top panel shows the DB1 pattern, and the bottom panel shows the DB2 pattern.

FIGURE S5 The putative DB homology structure for each MBFV. The RNA secondary structure was predicted online by Mfold (http://unafold.rna.albany.edu/?q = mfold) and was trimmed manually.

FIGURE S6 The proposed conserved secondary structure and sequence motifs within the 5′ and the 3′ ends of the representative *flavivirus* genomes. The nucleotide numberings are included.
