# Supplementary figures and images for: Universal RNA Secondary Structure Insight Into Mosquito-Borne Flavivirus (MBFV) cis-Acting RNA Biology
Source: Front Microbiol. 2020 Mar 27;11:473. doi: 10.3389/fmicb.2020.00473 (PMC7118588; doi:10.3389/fmicb.2020.00473)

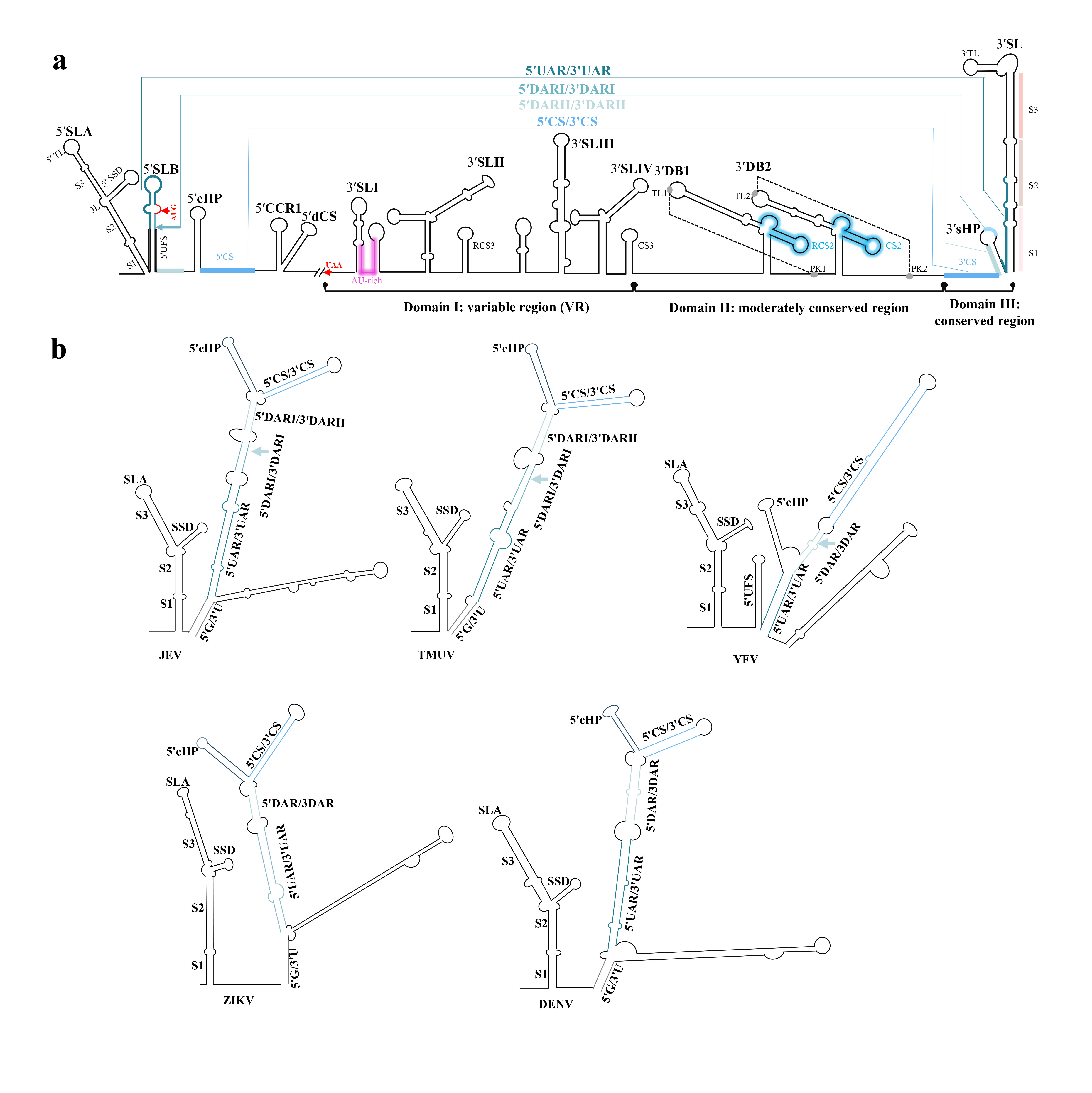

Supplement: FIGURE S1 — (a) Linear models of MBFV cis-acting RNA and the RNA-acting binding proteins (RBP) for MBFV. Using JEVG as model, the proposed secondary structures of the core cis-acting RNA represent the typical MBFV genomic RNA. Meanwhile, the conformations of individual cis-acting RNA secondary structure are annotated. (b) Summary of the representative MBFV 5′-3′cirRNA models. The minimum-free-energy structure is adopted. Colored lines indicate the interacting RNA elements involved in genome cyclization. The same color is used for equivalent structures in different MBFVGs. [file Image_1.JPEG]

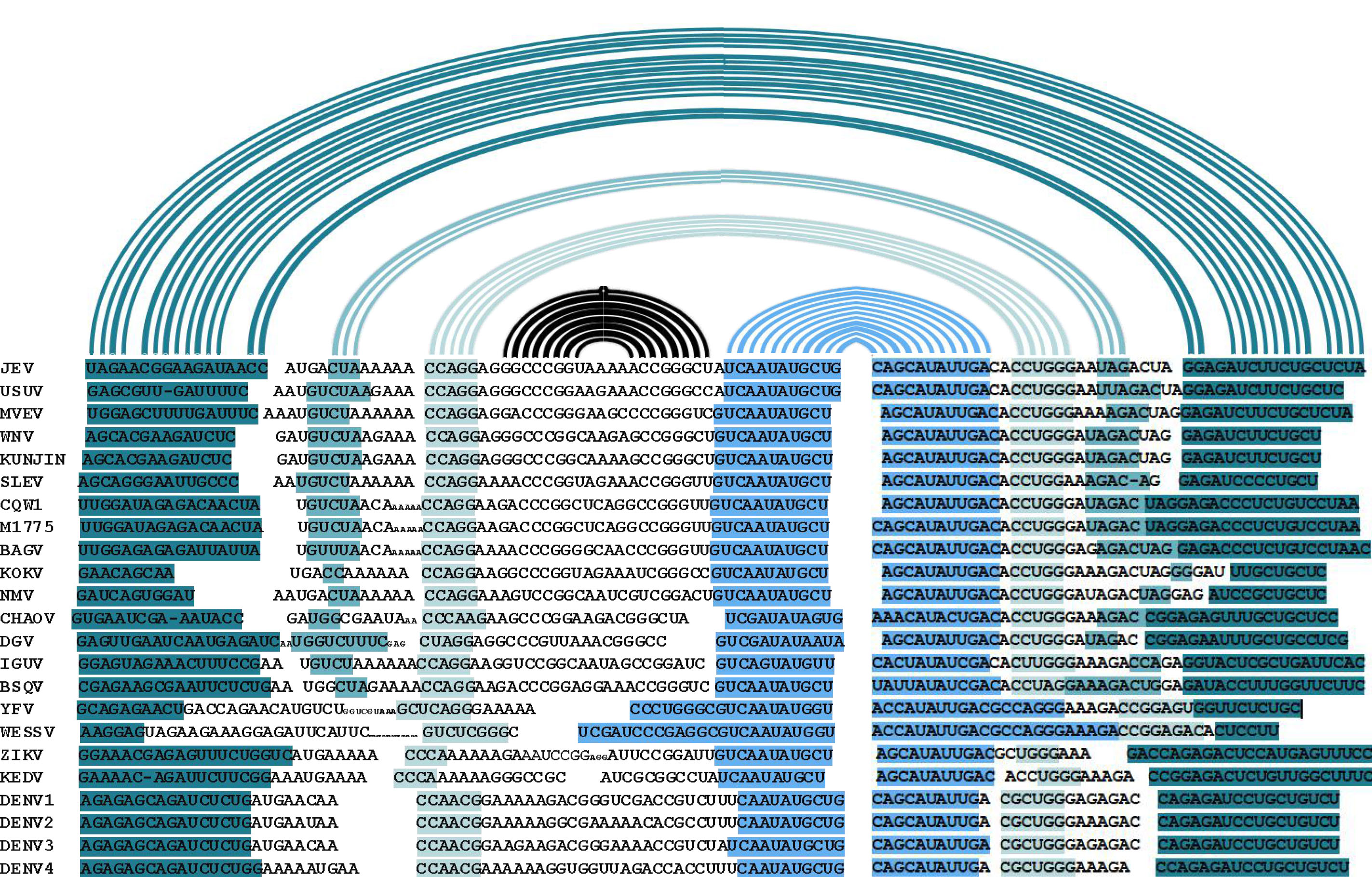

Supplement: FIGURE S2 — Comparison of the nucleotide sequences of 5′-3′cirRNA in MBFVs. The different colors denote the indicated inverted complementary sequences. Above the multiple alignments, the secondary structure of JEVG is shown using an arc plot. [file Image_2.JPEG]

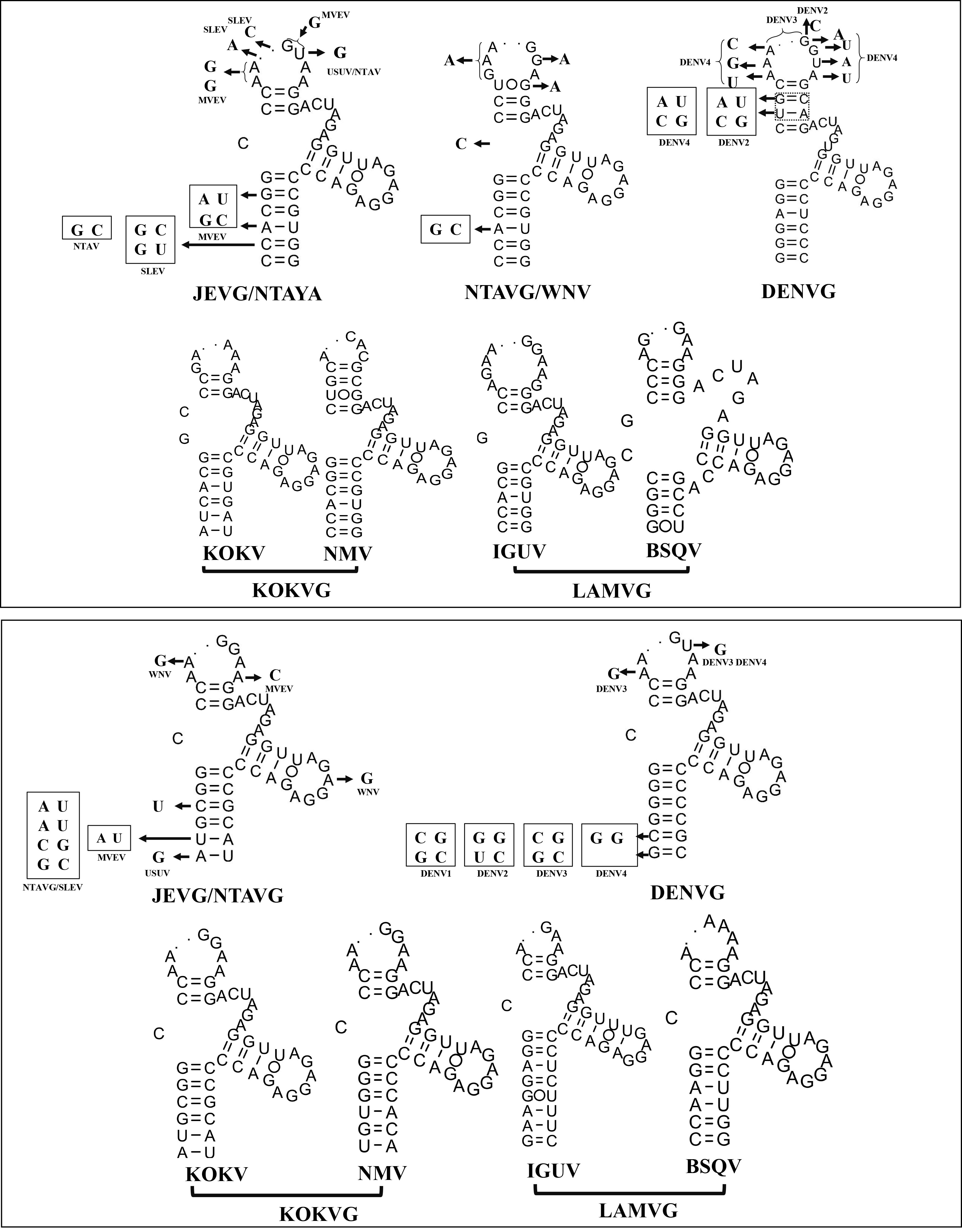

Supplement: FIGURE S4 — The conserved patterns of structural homology of dual-DB. Divergent nucleotides of group-specific DBs in dual-DB MBFVGs are mapped. The top panel shows the DB1 pattern, and the bottom panel shows the DB2 pattern. [file Image_4.JPEG]

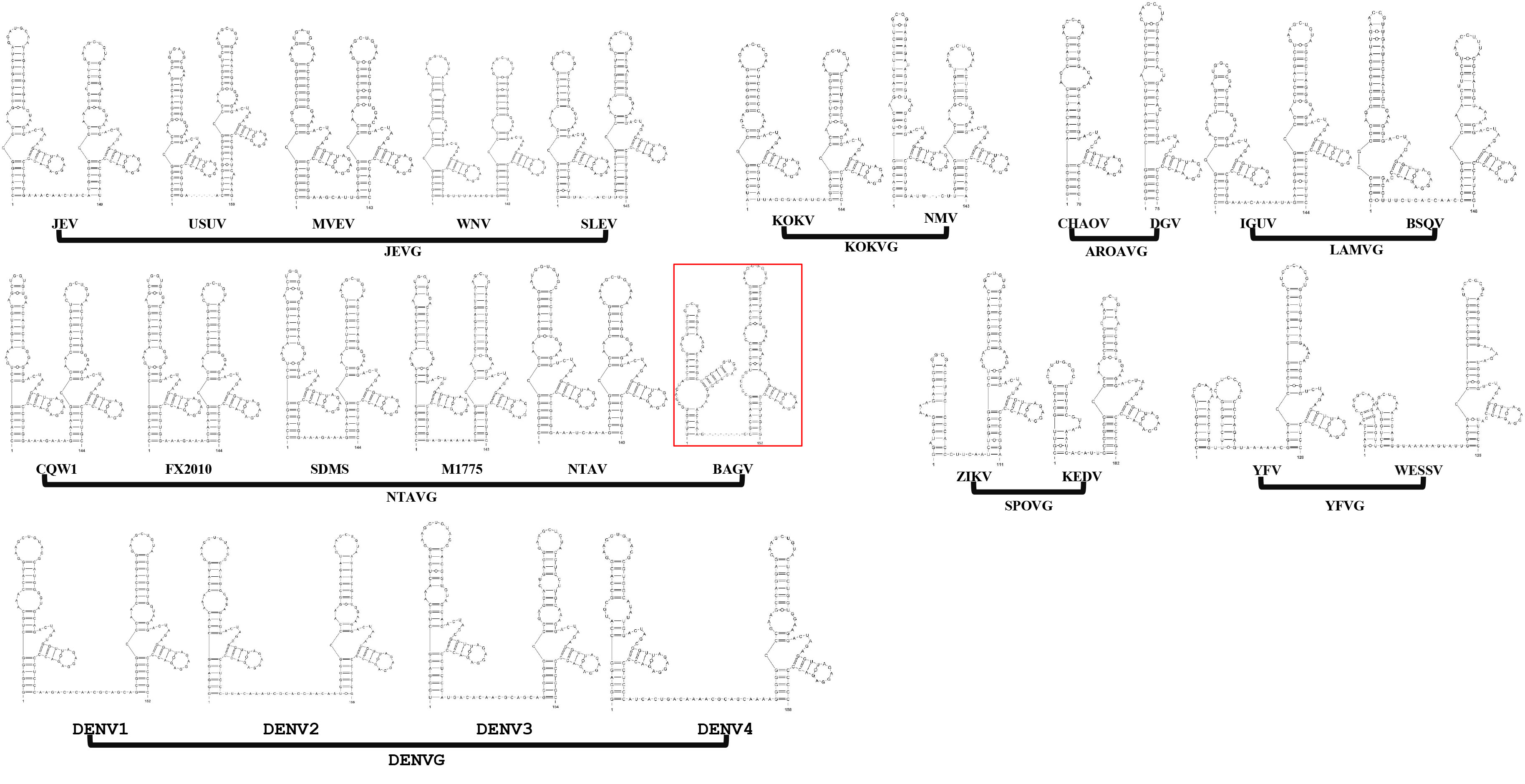

Supplement: FIGURE S5 — The putative DB homology structure for each MBFV. The RNA secondary structure was predicted online by Mfold (http://unafold.rna.albany.edu/?q = mfold) and was trimmed manually. [file Image_5.JPEG]

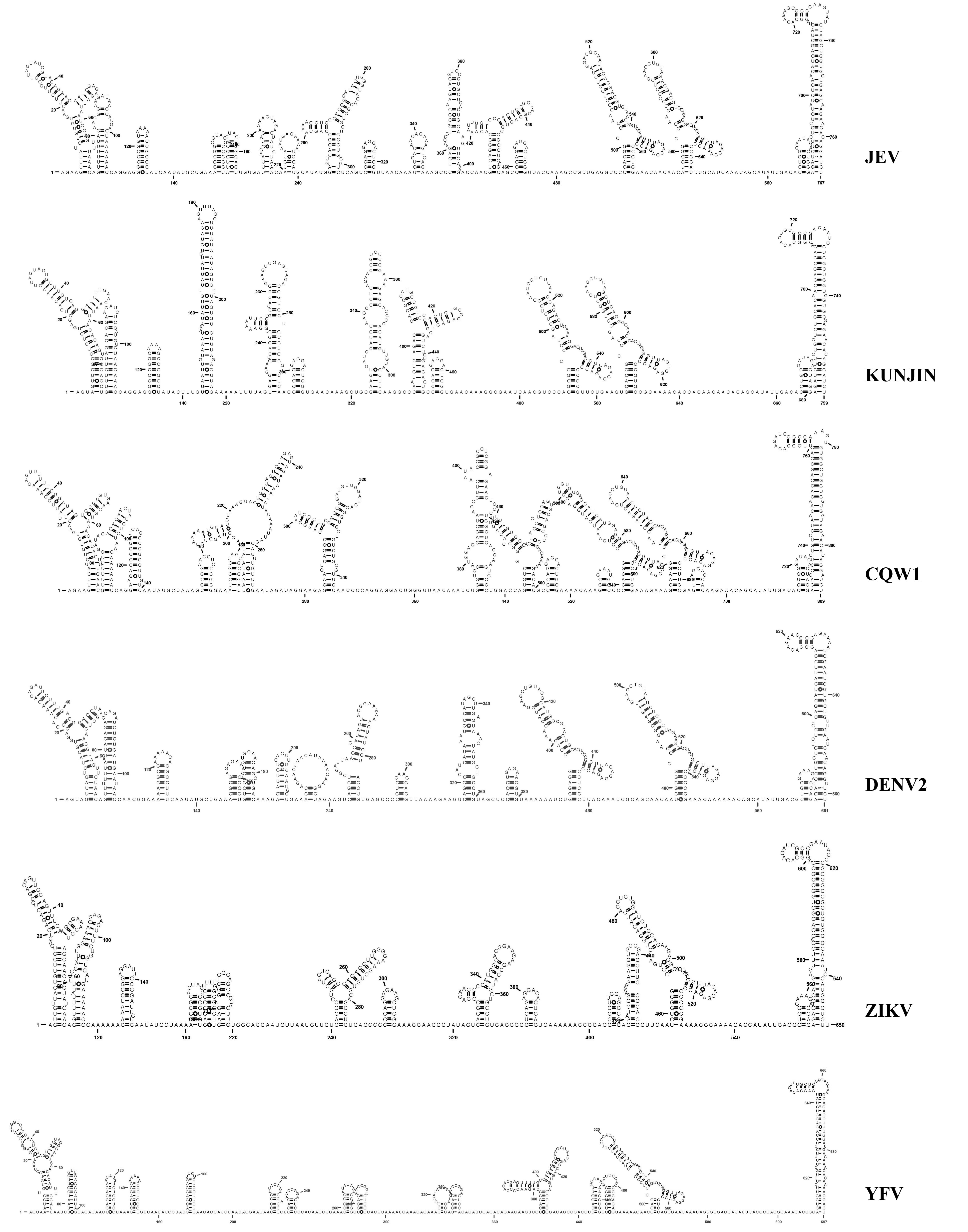

Supplement: FIGURE S6 — The proposed conserved secondary structure and sequence motifs within the 5′ and the 3′ ends of the representative flavivirus genomes. The nucleotide numberings are included. [file Image_6.JPEG]
